# Evaluation of the Prevalence of Barotrauma and Affecting Factors in Patients with COVID-19 during Follow-Up in the Intermediate Care Unit

**DOI:** 10.3390/jpm12111863

**Published:** 2022-11-07

**Authors:** Ayse Bahadir, Sinem Iliaz, Mehmet Hursitoglu, Gul Unalan, Sibel Yurt, Mehmet Akif Ozgul

**Affiliations:** 1 Department of Pulmonary Medicine, Basaksehir Cam and Sakura City Hospital, University of Health Sciences, Basaksehir, Istanbul 34480, Turkey; 2 Department of Pulmonology, Memorial Bahçelievler Hospital, Bahcelievler, Istanbul 34180, Turkey; 3 Internal Medicine Department, Bakırkoy Dr. Sadi Konuk Training and Research Hospital, Bakirkoy, Istanbul 34147, Turkey

**Keywords:** COVID-19, pneumothorax, barotrauma, mediastinal emphysema, pneumonia

## Abstract

It is known that pneumothorax (PX) and pneumomediastinum (PM) develop due to COVID-19 disease. The objective of our study was to determine the prevalence of PX/PM due to COVID-19 in the intermediate intensive care unit (IMCU) and to evaluate the factors causing barotrauma and also the clinical outcomes of these patients. A total of 283 non-intubated patients with COVID-19 pneumonia followed up in the IMCU in a 1-year period were included in the study. The patients were classified as group 1 (having barotrauma) and group 2 (without barotrauma). The rate of barotrauma was 8.1% (*n* = 23, group 1). PX developed on the right hemithorax in 12 (70.6%) patients. Group 1 had statistically significantly higher 28-day mortality rates compared with group 2 (*p *= 0.014). The eosinophil and d-dimer levels of the patients in group 1 were higher, while C-reactive protein (CRP), fibrinogen, and albumin levels were lower than Group 2 (*p* < 0.001, *p *= 0.017, *p *= 0.001, *p* < 0.001), and *p* < 0.001, respectively). The similar rates of NIMV administration in our study groups support that barotrauma is not the only mechanism in the development of PX/PM. The findings of high blood eosinophil count and low blood levels of CRP, albumin, and fibrinogen in the barotrauma group of our study might be a pathfinder for future studies.

## 1. Introduction

Pneumothorax (PX) develops as a result of a connection between the alveoli and the pleural space, or between the pleural space and the atmosphere, or gas accumulation due to gas-producing microorganisms in the pleura. If there is no underlying cause to identify, it is called primary PX. On the other hand, secondary PX occurs due to underlying pulmonary pathologies such as chronic obstructive pulmonary disease (COPD), asthma, cystic fibrosis, interstitial lung diseases (e.g., idiopathic pulmonary fibrosis [IPF], histiocytosis X, lymphangioleiomyomatosis [LAM], and sarcoidosis), infection (e.g., pneumocystis jiroveci pneumonia [PJP], tuberculosis [TB], and necrotizing pneumonia), connective tissue diseases, and cancer [1].

PX and pneumomediastinum (PM) are known to associate with pulmonary involvement in coronavirus disease 2019 (COVID-19), caused by severe acute respiratory syndrome coronavirus 2 (SARS-CoV-2) [2,3,4]. Such life-threatening complications lead to prolonged hospitalizations and increased healthcare costs. In meta-analyses examining PX/PM cases due to COVID-19, the rate of barotrauma in patients admitted to the emergency department is reported as between 0.3 and 1.4%. That rate increases up to 12.8–23.8% in patients requiring invasive mechanical ventilation (IMV) [2,3,4].

As a result of studies in the literature, high-flow nasal cannula (HFNC) and non-invasive mechanical ventilation (NIMV) have become the first choice in the treatment of severe respiratory failure due to COVID-19 in both intensive care units (ICU) and wards [5]. Long-term use of these methods, which are effective in avoiding intubation, may cause PX/PM. Different incidence rates of PX/PM due to HFNC and NIMV use in COVID-19 were reported in the literature due to small sample sizes, design differences, and incomplete data in the studies [2,6]. PX/PM due to COVID-19 occurs both in ventilated and non-ventilated patients. Thus, related studies show that factors other than barotrauma are also effective in the pathogenesis of PX/PM. However, the exact pathophysiology of COVID-19 infection leading to PX/PM is yet to be determined and not clearly established [3,7]. In a meta-analysis examining the development of spontaneous PX in non-ventilated patients, it was reported that parameters such as clinical findings, medications, type and quantity of oxygen support, and NIMV were heterogeneous [8]. Therefore, in the present study, we included patients who were followed in the intermediate care unit (IMCU) and received NIMV or HFNC due to COVID-19. The primary objective of our study was to determine the prevalence of PX/PM due to COVID-19 in the IMCU. The secondary objective was to evaluate the factors causing PX and PM, and also the clinical outcomes of these patients.

## 2. Materials and Methods

### 2.1. Design and Inclusion/Exclusion Criteria

The study included all non-intubated adult patients aged 18 years and older with COVID-19 who were hospitalized in the IMCU between January 2021 and December 2021. The patients were diagnosed as having COVID-19 through polymerase chain reaction (PCR) test positivity, or in PCR-negative cases, with clinical radiologic compatibility. The cases presented with newly onset acute respiratory failure without other known etiology or any culture positivity at presentation, with bilateral patchy peripheral ground glass infiltration or having close contact with COVID-19 disease in the previous week of presentation were included as PCR-negative subjects. According to the Turkish Ministry of Health guidelines, patients with COVID-19 and tachypnea (≥30/minute) and SpO2 levels below ≤90% in room air, and bilateral diffuse pneumonia findings on chest X-ray or tomography are defined as having “severe pneumonia”. In addition, it is recommended to follow patients with SpO2 < 90% or PaO2 < 70 mm Hg despite 5 L/min oxygen therapy in the ICU/MICU [9]. In the study period, 314 patients met these criteria. Patients who developed PX or PM ± subcutaneous emphysema (SCE) during follow-up in the IMCU were recorded and classified as the barotrauma group (group 1). Twenty-seven patients were excluded from the study because they developed PX/PM ± SCE while in the ward or intubated or had missing data on follow-up. Also, three pregnant patients and a patient who developed PX due to TB were excluded from the study. The study group was composed of 283 patients. Demographic characteristics, radiologic findings (posteroanterior [PA] chest X-ray and thorax computed tomography [CT]), respiratory support type (HFNC/NIMV), in-hospital and 28-day mortality, and IMCU length of stay were recorded retrospectively. Laboratory findings (e.g., complete blood count, C-reactive protein [CRP], procalcitonin [PCT], biochemistry, COVID-PCR result) of the patients on the first day of hospitalization in the IMCU were recorded. The neutrophil-lymphocyte ratio (NLR) was calculated by dividing the absolute neutrophil count by the lymphocyte count. The systemic inflammation index (SII) index was calculated as “peripheral platelet count (plt) × N/L” (SII = plt × N/L).

Patients were divided into two radiologic severity groups according to the presence of ground-glass consolidation of more than 50% of the total lung area or less on the arrival thorax CT/PA chest X-ray. During the IMCU follow-up, the patients underwent PA chest X-ray imaging every 1–3 days or when their clinical condition worsened.

### 2.2. Treatment Protocol

All patients were treated with antiviral (favipiravir) treatment according to the standard Turkish Ministry of Health COVID-19 protocol (2 × 1600 mg on the 1st day, 2 × 600 mg on the next 4 days) [9]. Pulse methylprednisolone of 250 mg/day was administered intravenously (iv) for 3 days to patients followed in the IMCU whose SpO2 was <90% despite oxygen therapy and who were not in the first week of COVID symptoms. After the pulse steroid, treatment continued with 40 or 80 mg/day of methylprednisolone according to the clinical condition of the patients. According to the guideline, venous thromboembolism prophylaxis was performed on all hospitalized patients as long as they did not have active bleeding and thrombocytopenia (≤25–30,000 /µL). Enoxaparin sodium was administered 2 × 6000 IU subcutaneously (sc) to those with D-dimer values ≥1000 µg/L. If D-dimer was <1000 µg/L, then we gave 2 × 4000 IU of enoxaparin sodium sc. In the condition that the glomerular filtration rate (GFR) was 30–50, the enoxaparin dose was 1 × 4000 IU sc. If the GFR was <30, standard heparin was given. Anti-cytokine drugs were administered to patients who did not respond to glucocorticoid therapy for at least 3 days and whose signs of inflammation continued, or who progressed very rapidly and developed severe macrophage activation syndrome (MAS). An interleukin-6 (IL-6) receptor antagonist (tocilizumab) and IL-1 antagonist (anakinra) were randomly used in patients with suspected MAS (increased CRP, ferritin, D-dimer values and/or decreased lymphocyte, platelet counts and normal procalcitonin values). Tocilizumab was administered as a single dose of 400 mg/day iv at a dose of 8 mg/kg (maximum 800 mg). Anakinra was administered 200 mg iv three times a day for 3 days. For the following 3 days, the dose was 2 × 200 mg iv, and then 1 × 200 mg iv for 4 days. Thus, anakinra was given with a dose reduction for 10 days.

### 2.3. Oxygen and Respiratory Support

In cases where oxygenation could not be improved with conventional low flow methods (<15 L/min), HFNC (Hamilton-C3, Swiss) treatment was given. Nasal high-flow oxygen therapy was administered at a temperature of 34 °C, increasing the flow (maximum 60 L/min) to get SpO_2_ > 90% and ensuring FiO_2_ < 60%. NIMV (Hamilton-C3, Swiss) was administered via an oronasal mask to patients with SpO_2_ < 90% despite HFNC support. There were missing data on NIMV settings and they were not recorded because NIMV settings changed frequently according to the clinical conditions of the patients and the experience of the medical staff.

### 2.4. Outcomes

Patients who experienced PX/PM ± SCE (barotrauma) during follow-up in the IMCU were classified as “group 1” and those without barotrauma were classified as “group 2”. The duration of IMCU follow-up, which took until discharge to the ward, was recorded as the length of stay. Mortality rates of in-hospital stay and the first 28 days of IMCU were recorded.

### 2.5. Statistical Analysis

The Statistical Package for the Social Sciences (SPSS) version 22.0 for Windows software (IBM SPSS Statistics Data Editor, Chicago, IL, USA) was used for statistical analysis. Descriptive data are given as the number (*n*) of participants and frequency. Categorical variables are expressed as the number of patients (*n*) and the percentage value (%). A comparison of categorical variables was performed using the Chi-square and Fisher’s exact tests. Continuous variables are given as mean (with standard deviation) or as median (with interquartile range [IQR]) based on the normality of distribution. The Shapiro-Wilk test was used to determine whether the continuous variables were normally distributed. For continuous variables, Student’s *t*-test and the Mann-Whitney U test were used relative to the normality of distribution of the variables. Statistically significant findings were evaluated using multiple logistic regression analyses to determine the risk factors affecting the development of PX/PM. A *p*-value of <0.05 or a 95% CI for risk evaluation was considered statistically significant. COX regression analysis was performed in order to evaluate factors related to mortality. For the degree of correlation, The Phi and Cramer’s V value was determined (Range 0.0–1.0). A Phi and Cramer’s V value close to 0.00 indicates no association. A value > 0.15 is indicative of a strong, and >0.25 is indicative of a very strong association [10]. The sample size of our cohort includes all patients admitted during the study period. Considering the previous studies, the incidence of pneumothorax in the population is accepted as 14/100,000. When statistical significance is accepted as *p* < 0.05 and a 95% CI, to have a power of 0.80, there should be 185 patients in total. The study was undertaken in accordance with the principles of the Helsinki Declaration. All subjects gave informed consent to take part in the study. The study was approved by the Institutional Board Ethics Committee (Approval number: 2022-152 Basaksehir Cam and Sakura City Hospital).

## 3. Results

**Figure 1:**
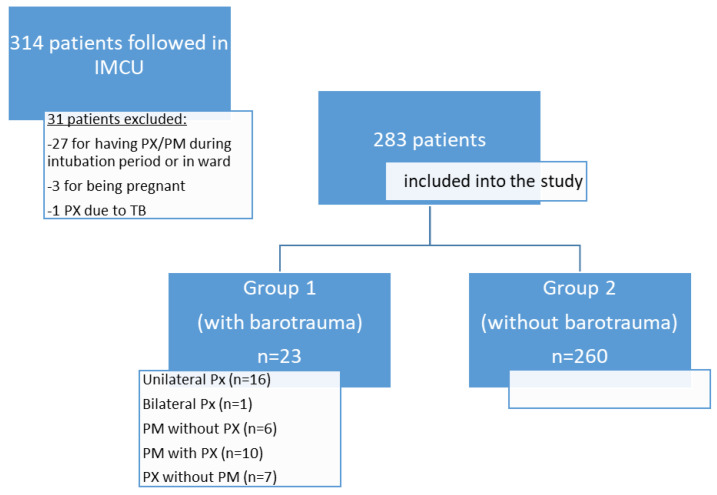
Flowchart of the study (IMCU: intermediate intensive care unit, PX: pneumothorax, PM: pneumomediastinum, TB: tuberculosis).

Among the 283 patients included in the study, the rate of barotrauma detected during IMCU follow-up was 8.1% (*n* = 23) (Figure 1). The comparison of characteristics related to demographic, laboratory, radiologic findings, and COVID diagnoses of groups 1 and group 2 is given in detail in Table 1 and Table 2.

### 3.1. Barotrauma

We detected barotrauma in 23 patients. PX developed in the right hemithorax in 12 (70.6%) patients out of 17 patients with PX ± PM, and it was detected on the left side in four patients (23.5%) and bilaterally in one patient (5.9%). PX alone was detected in seven patients and PM ± SCE alone in six patients; PX+PM was found together in 10 patients. Barotrauma developed within the first 10 days of IMCU admission in 14 (60.9%) patients, and after 10 days of IMCU stay in nine (39.1%) patients. Barotrauma was diagnosed in CT in two patients and PA chest X-ray in the remaining 21 patients. Tube thoracostomy was performed on 10 patients (43.5%) in group 1, and conservative treatment was given to 13 (56.5%) patients because barotrauma findings were minimal or due to the presence of PM without PX.

### 3.2. Demographic Characteristics

There was no statistically significant difference between groups 1 and 2 in terms of mean age, sex, comorbidity, and underlying lung disease (*p >* 0.05 for all, details in Table 1). The most common comorbidity was hypertension (HT) with a rate of 38.9% and diabetes mellitus (DM) at a rate of 31.4%. There was no statistical difference in the development of barotrauma in terms of DM or HT coexistence (*p *= 0.913 and *p *= 0.379, respectively). The rate of underlying lung disease among all study groups was 20.8% and the most common respiratory diseases were asthma (8.2%) and COPD (7.4%). No underlying lung disease was found in 87% of group 1 (Table 1).

### 3.3. Radiology

The rate of COVID PCR positivity was 84.5%. There was no statistically significant difference between the groups in terms of the severity of radiologic involvement due to COVID-19 and barotrauma (*p *= 0.778) (Table 1). When we compared the radiological severity on the day of PX/PM with initial imaging, chest X-ray showed progression in severity in 44.4% of the barotrauma group and the chest X-ray were stable in 55.6% of the Group 1.

### 3.4. Laboratory Findings

There was no significant difference between the total leukocyte (WBC), neutrophil, lymphocyte, plt counts, hemoglobin, PCT, ferritin, lactate dehydrogenase (LDH), fasting blood glucose, GFR results, and barotrauma groups. The eosinophil and D-dimer levels of the patients in group 1 were higher, and the CRP, fibrinogen, and albumin levels were lower than in group 2 (*p* < 0.001, *p *= 0.017, *p *= 0.001, *p* < 0.001, and *p* < 0.001, respectively). No significant difference was found between NLR and SII levels (*p *= 0.706 and *p *= 0.480, respectively) (Table 2).

### 3.5. Treatment

Although there were no differences between the average daily steroid and LMWH doses of groups 1 and 2 (*p *= 0.162 and *p *= 0.285, respectively), the number of days on steroid and LMWH were higher in group 1 (*p* < 0.001 and *p *= 0.015, respectively). There were no differences between the groups in terms of the use of tocilizumab and anakinra (*p *= 0.795 and *p *= 0.444, respectively) (Table 3).

### 3.6. Respiratory Support

Among the total 283 patients, 88.7% had HFNC support, and 46.3% were treated with NIMV. Barotrauma developed in 6.8% of patients receiving HFNC and 7.6% of patients receiving NIMV. There was no difference between the rates of using NIMV in groups 1 and 2, but HFNC use was found to be statistically significantly less in group 1 (*p *= 0.778 and *p *= 0.020, respectively). There were no differences in mean flow rates of HFNC between groups 1 and 2 (*p *= 0.942) (Table 3).

### 3.7. Outcomes

Group 1 had a statistically significantly longer length of IMCU stay and higher in-hospital and 28-day mortality rates compared with group 2 (*p* < 0.001, *p *= 0.008, and *p *= 0.014, respectively). Ouf of 23 patients with barotrauma, 15 patients (65.2%) ended up getting invasive ventilation in ICU. The 28-day mortality risk of patients in group 1 was 3.15 times higher than in group 2 (73.7% vs. 47.3%) (odds ratio [OR]: 3.15, 95% CI: [1.2–8.2]; *p *= 0.014). Details are given in Table 3. Comparing barotrauma presence with the mortality with the Chi-square test yielded a significant correlation (*p* < 0.05). For the degree of correlation, The Phi and Cramer’s V coefficient was 0.16. According to these test results, there was a strong correlation between the barotrauma presence and the outcome. In COX regression analysis to evaluate factors related to mortality, having barotrauma, any comorbidity, or any gender were not statistically significant. There was no factor affecting mortality except advanced age and high CRP (Odds ratio > 1 and *p* < 0.05 for both). In addition, tube-thoracostomy was performed in 10 patients out of 23 patients with barotrauma. Nine patients (%90) out of 10 patients with intervention and also nine patients (69.2%) out of 13 patients without an intervention for barotrauma were dead. The difference between groups was not statistically significant (*p *= 0.33).

For the characteristics found to be statistically significantly different between groups 1 and 2, we performed binary logistic regression analysis. According to the logistic regression analysis, albumin, fibrinogen, eosinophil, and CRP levels were found to affect the occurrence of barotrauma, but no D-dimer effect was found (Table 4). Also, the duration of HFNC, steroid, and LMWH use was not statistically significant.

## 4. Discussion

In our study, we examined the clinical and laboratory characteristics of patients in our hospital’s IMCU who developed barotrauma while on HFNC and NIMV support due to COVID-19 disease-induced respiratory failure. We found that non-intubated patients who developed PX/PM while being followed up in the IMCU had a poorer prognosis, higher first 28-day mortality rate, and longer hospital stay than patients without PX/PM. Our results showed that these findings were not related to age, sex, or the radiologic severity of the disease.

Although the rate of COVID-19–related PX/PM was 0.9% among patients who were admitted to the emergency department, its prevalence was higher than in the normal population. Additionally, the average age was higher than the normal population and the rate of accompanying PM and SCE to PX was more frequent [11]. The rate of PX/PM was 1.4–2% among hospitalized (non-ICU medical wards) patients with COVID-19. However, this rate increased up to 6.1–15% in patients followed up in the ICU. In addition, barotrauma was detected in 9–35.7% of patients receiving NIMV, 17–23% of patients receiving HFNC, and 15.1% of patients receiving ordinary nasal or mask oxygen. In previous studies, the mean age of patients with barotrauma was between 60–80 years in half of the patients, and the vast majority (72.5–82%) were male [3,4,6,11,12,13]. Considering patients who were followed up on IMV for severe pneumonia in previous years, the rate of barotrauma in SARS and Middle East respiratory syndrome (MERS) was 12–34% and 30%, respectively.

Regarding barotrauma due to COVID-19, the rate of barotrauma in patients on IMV has been reported as 15–30% [2]. In an ICU-based study, the rate of barotrauma was 13% in patients with pneumonia due to COVID-19 and receiving IMV [13]. Compared with that, the rate of barotrauma in our non-intubated patients in the IMCU was lower (8.3%). The rate of unilateral and right PX was also high in our study, in accordance with the literature [12,14]. In addition, the sex and age distributions of our study group with barotrauma were compatible with the literature [6,11,12,13,14].

It has been hypothesized that interstitial emphysema develops as a result of hyperinflation and alveolar pressure increase due to cough and airway obstruction in COVID-19, and PX/PM occurs with air leaking into the mediastinum along the bronchoalveolar pathway (Macklin effect). Although the symptom duration is thought to support hyperinflation, immunologic mechanisms are also blamed for the development of PX/PM. It is suggested that lymphocytes gather from the periphery to the lung and cause alveolar damage. Accordingly, lymphopenia is known as a poor prognostic factor [12,15]. On the ground of alveolar damage, the increase in intrathoracic pressure resulting from coughing or positive pressure ventilation contributes to the development of PX/PM [2,3,4]. Conflictingly, another study found no relationship between the development of PX/PM and cough [11]. We cannot interpret its contribution to the development of PX/PM because symptom questioning was not recorded in our study. In addition, HFNC and NIMV are used as safe and effective methods to avoid IMV in patients with acute hypoxemic respiratory failure due to COVID-19 [16].

In our study, no difference was found between groups 1 and 2 in terms of NIMV application frequency, but the rate of HFNC use in group 1 was lower than in group 2. It is known that the applied heated and humidified gas reduces the dead space, provides a positive end-expiratory pressure (PEEP) effect in the alveoli, and reduces the work of breathing [17]. The low rate of HFNC use in the barotrauma group in our study suggested that the positive effect of HFNC on respiratory workload might reduce the risk of barotrauma by reducing hyperinflation. These findings also support that barotrauma is not the only mechanism in the development of PX/PM in COVID-19. Similarly, it is known that recruitment maneuvers and prone positioning in ARDS increase the susceptibility to barotrauma by increasing alveolar pressure and neuromuscular blockade (NMB) by causing pleural pressure changes [18]. Intubated patients were not included in our study. Therefore, no recruitment maneuver was performed and NMB was not used. We cannot interpret the contribution of these mechanisms to the development of barotrauma because our records of the frequency and duration of the applied prone position therapy were not included in the study. Contrary to the higher rate of underlying lung disease in spontaneous pneumothorax in non-COVID patients, there was no underlying primary lung pathology in the patients with COVID-19 in general. This information suggests that PX/PM is seen due to damage to the lung by SARS-CoV-2 itself [1,12,19]. Consistent with the literature, approximately 80% of our patients with PX/PM had no underlying lung disease.

It is known that D-dimer and fibrinogen levels increase, and prothrombin time (PT) and activated prothrombin time (aPTT) are prolonged in patients with a high risk of arterial and venous thrombus due to COVID-19 [20]. Bronchial occlusion and microthrombi are seen as a result of alveolar damage and fibromyxoid exudate accumulation due to COVID-19, which may contribute to the development of PX/PM by paving the way for cyst formation [11,14,21,22]. In addition, microthrombi were also detected in lung specimens examined after surgery for prolonged air leakage due to PX [3]. We performed venous thrombus prophylaxis on our patients during the follow-up in the IMCU. However, D-dimer levels in group 1 were found to be statistically significantly higher than in group 2. Although this supports the barotrauma-microthrombus relationship, we cannot reach a definite conclusion about the presence of embolism/thrombus because we could not evaluate our patients with thorax CT angiography.

Inflammation, hyperferritinemia, hemodynamic instability, multi-organ failure, and high inflammatory response due to cytokine storm in patients with severe COVID-19 infection are thought to cause alveolar lung damage [23]. Although its effect was not known in the early stages of the pandemic, in the recent RECOVERY study, dexamethasone was shown to reduce 28-day mortality compared with standard treatment in patients receiving IMV or oxygen support [24]. In addition, it has been reported that the use of tocilizumab (an IL-6 receptor antagonist) and anakinra (an IL-2 receptor antagonist) in patients who develop cytokine storm reduced mortality in patients with respiratory failure [25]. In our study, there was no difference in daily steroid doses in the groups based on the presence of barotrauma, but the cumulative steroid dose of the barotrauma group was higher (Table 3). This may be due to the longer hospital stay in the barotrauma group. Although approximately half of the patients used tocilizumab or anakinra in total, no statistically significant difference was found based on the use of anti-cytokines between the groups with and without barotrauma. In a study including intubated patients, no difference was found in tocilizumab treatment and cumulative steroid doses between the barotrauma group and the non-barotrauma group [14]. The mortality rate in the patients with PX/PM in COVID-19 was also evaluated in the literature. In Bonato’s study, similar to our study, an increase in in-hospital and 90-day mortality and a prolonged length of hospital stay were found in the barotrauma group [12]. In Ekanem et al.’s study, all hospitalized patients (*n* = 1619) with COVID-19 were analyzed and the spontaneous PX/PM rate was 1.4%. In the same study, 28-day mortality was 36% among patients with barotrauma [4]. In our study, the mortality rate was also high in the barotrauma group (51.9%). It was even higher than the above study’s rate. We think that the patient population having severe COVID-19 pneumonia in the IMCU, and the fact that the vaccination program was not maintained effectively in our country at that time may have contributed to the higher mortality rate among our patients. Although advanced age and high serum CRP levels were risk factors for mortality. The barotrauma patients’ group was younger and had lower serum CRP levels than the non barotrauma patients’ group. These factors could not explain the higher mortality rate in the barotrauma group patients. Additionally, the Phi and Cramer’s V coefficient for this association was 0.16 (strong association) [10]. Hence, it seems that the presence of barotrauma is an independent risk factor for the bad outcome in our study. Large scaled studies are still needed to confirm this finding, though. When the factors affecting the development of barotrauma were evaluated using logistic regression analysis, low fibrinogen, high eosinophil, albumin, and CRP levels were related to PX/PM, but no D-dimer effect was found (Table 4). In our study, the low serum CRP levels in the barotrauma group were a surprising finding. In this retrospective study, we took the serum CRP on the first day of hospitalization as a reference in the analysis. Taking several measures in proceeding hours or days might give more information in this issue. In a study by Mahrous et al., the survived acute lung injury (ALI) patients had unexpectedly higher serum CRP levels than the died ALI patients [26]. Could this be the case in our study? This also needs to be studied further. Eosinophils, which have pro-inflammatory, immunoregulatory, and antiviral properties, decrease secondary to acute inflammation in serious diseases such as sepsis. Also, low eosinophils were reported as a poor prognostic factor and an increase in their number is accepted as a sign of clinical improvement in a severe disease course [27,28]. It is also known that after spontaneous PX, the number of leukocytes and eosinophils in pleural effusion and peripheral blood increase secondary to pleural inflammation [29]. In our study, the increase in eosinophils in the barotrauma group can be evaluated in this way.

The limitations of our study may be listed as the single-center, retrospective nature, and the low number of barotrauma cases. In addition, our study could not give an average pressure value leading to barotrauma because the NIMV settings changed frequently depending on the clinical status of the patients. However, most studies evaluated barotrauma in intubated patients in the ICU, but in our study, all of our patients were non-intubated and consisted of those who developed barotrauma while being followed up with HFNC/NIMV. We should mention that all of our patients had severe pneumonia due to COVID-19 and the usage rate of anti-inflammatory agents such as steroids and anti-cytokines was quite high. Unfortunately, we could not perform a receiver operating curve (ROC) analysis for the laboratory-determined risk factors of barotrauma in our study (eosinophil, CRP, albumin, and fibrinogen). The main reason is the low sample size of the barotrauma group. Nevertheless, we believe that these parameters will guide similar future studies.

## 5. Conclusions

In conclusion, the prevalence of barotrauma in our non-intubated patients with COVID-19 in the IMCU was in line with the results of previous studies. It could be a sign of a bad outcome. The findings of high blood eosinophil count and low blood levels of CRP, albumin, and fibrinogen in the barotrauma group of our study would be a pathfinder for future studies in this field. However, until proven otherwise, we think that these simple and routinely used predicting blood parameters should be used in daily practice. It may help in the early detection and management of this fatal and life-threatening complication. In addition, the similar rates of NIMV administration in our study groups support that barotrauma is not the only mechanism in the development of PX/PM.

## Figures and Tables

**Table 1:** Comparison of demographic characteristics of the groups according to the presence of barotrauma.

(*n*/%)	Total (*n* = 283)	Group 1 (*n* = 23)	Group 2 (*n* = 260)	*p*-Value
Age (mean ± SD)	63 ± 14.8	56.5 ± 14.0	62.5 ± 14.8	0.062
Sex (male)	175 (61.8%)	16 (69.6%)	159 (61.2%)	0.506
Comorbidity (+)	205 (72.5%)	15 (65.2%)	190 (73.4%)	0.464
Diabetes mellitus (+)	89 (31.4%)	7 (30.4%)	82 (31.5%)	0.913
Hypertension (+)	110 (38.9%)	7 (30.4%)	103 (39.8%)	0.379
Underlying lung disease (+)	63 (22.3%)	3 (13%)	60 (23%)	0.430
COVID PCR (+)	84.5 %	82.6%	84.6%	0.760
Radiologic severity (>50% involvement +)	159 (56.4%)	12 (52.2%)	147 (56.7%)	0.778

SD: Standard deviation.

**Table 2:** Comparison of laboratory characteristics of the groups according to the presence of barotrauma.

Mean ± SD or Median (IQR) *	Total (*n* = 283)	Group 1 (*n* = 23)	Group 2 (*n* = 260)	*p*-Value
WBC (10^3^/µL)	11.1 ± 6.4	13.4 ± 6.0	10.8 ± 6.5	0.104
Neutrophils (10^3^/µL)	9.7 ± 6.3	11.9 ± 6.0	9.4 ± 6.3	0.909
Lymphocytes (10^3^/µL)	0.89 ± 1.2	0.85 ± 0.5	0.90 ± 1.3	0.917
NLR (IQR)	12 (7.14–21.7)	13 (7.4–37)	12 (7–21)	0.706
Eosinophils (10^3^/µL)	0.01 ± 0.6	0.06 ± 0.16	0.01 ± 0.0	**<0.001**
Hemoglobin (g/dL)	12.2 ± 2.3	12.8 ± 2.4	12.1 ± 2.3	0.328
Platelets (10^5^/µL)	248 ± 107.9	259.1 ± 90.9	247.8 ± 109.4	0.631
SII (IQR)	2902 (1414–5468)	3115 (1602–5486)	2877 (1400–5452)	0.480
CRP (IQR) mg/L	96 (55–167)	59 (5.4–103.5)	101 (59–168)	**0.001**
PCT (µg/L)	1.85 ± 7.0	1.17 ± 0.3	1.15 ± 0.3	0.577
Fibrinogen (mg/dL)	587 (440–700)	414 (224–557)	599 (461–706)	**<0.001**
Ferritin (IQR) (µg/L)	816 (435–172)	2585.5 ± 7062.2	1194.8 ± 1359.8	0.341
Albumin (IQR) (mg/dL)	33 (30–36)	30 (25–33)	33 (30–36)	**<0.001**
LDH (IQR) (U/L)	474 (342–629)	515 (374–731)	467 (340–628)	0.290
FBG (mg/dL)	185.2 ± 90.1	186.1 ± 77.5	185.1 ± 91.2	0.961
BUN (mg/dL)	63.6 ± 43.8	62.4 ± 4.51	63.8 ± 43.8	0.891
Creatinine (mg/dL)	1.2 ± 1.1	0.85 ± 0.67	1.28 ± 1.22	0.097
GFR (mL/min/m^3^)	75.8 ± 34.0	86.0 ± 38.4	74.9 ± 33.5	0.133
D-dimer (IQR) (mg/L)	1.1 (0.60–3.2)	3.5 (0.8–8.3)	1.0 (0.6–2.7)	**0.017**

SD: Standard deviation, WBC: White blood cell, NLR: neutrophil to lymphocyte ratio, IQR: interquartile range, SII: systemic inflammation index, CRP: C reactive protein, PCT: procalcitonin, LDH: lactate dehydrogenase, FBG: fasting blood glucose, BUN: blood urea nitrogen, GFR: glomerular filtration rate. * For values not distributed normally, we gave the median and interquartile range. Significant *p*-values are given in bold.

**Table 3:** Treatment and outcome-related characteristics of the groups according to the presence of barotrauma.

Mean ± SD or Median (IQR) *	Total (*n* = 283)	Group 1 (*n* = 23)	Group 2 (*n* = 260)	*p*-Value
**Treatment**				
Having CS treatment (*n*/%)	263 (92.9%)	21 (91.3%)	242 (94.9%)	0.600
Daily CS * dosage (mg/day)	55.7 ± 47.1	41.9 ± 20.8	56.8 ± 48.4	0.162
Duration of CS (days)	15.1 ± 9.7	22.1 ± 16.0	14.5 ± 8.8	**<0.001**
Daily LMWH dosage (IU/day)	10,487 ± 2598	10,409 ± 2966	11,043 ± 2549	0.285
Duration of LMWH (day)	15.4 ± 10.9	20.7 ± 16.1	14.9 ± 10.2	**0.015**
Having anakinra treatment (*n*/%)	79 (27.9%)	8 (34.8%)	71 (27.3%)	0.444
Having tocilizumab treatment (*n*/%)	118 (41.7%)	9 (39.1%)	109 (41.9%)	0.795
**Respiratory support**				
Having HFNC treatment (*n*/%)	251 (88.7%)	17 (73.9%)	234 (93.2%)	**0.020**
HFNC flow rate (L/min)	57.1 ± 9.9	57.0 ± 6.5	57.1 ± 10.1	0.942
Having NIMV treatment (*n*/%)	131 (46.3%)	10 (43.5%)	121 (46.7%)	0.778
**Outcome**				
Length of hospital stay (day)	12.1 ± 8.8	19.1 ± 11.7	11.5 ± 8.3	**<0.001**
In-hospital mortality (*n*/%)	147 (51.9%)	18 (78.3%)	129 (49.6%)	**0.008**
First 28-day mortality (*n*/%)	140 (49.5%)	17 (73.7%)	123 (47.3%)	**0.014**

CS: corticosteroid, CS *: given according to methylprednisolone doses, LMWH: low-molecular-weight heparin, HFNC: high-flow oxygen cannula, NIMV: non-invasive mechanical ventilation. Significant *p*-values are given in bold.

**Table 4:** Evaluation of factors affecting the development of barotrauma with logistic regression analysis.

	OR	*p*-Value	95% CI (Min–Max)
Albumin	0.878	0.002	0.841–0.917
Fibrinogen	0.996	0.019	0.994–0.998
Eosinophils	3.528	0.006	1.026–6.029
D-dimer	1.009	0.856	0.958–1.062
CRP	0.992	0.049	0.988–0.996

OR: odds ratio, CRP: C-reactive protein, Significant *p*-values are given in bold.

## Data Availability

The data presented in this study are available on request from the corresponding author. The data are not publicly available due to privacy reasons.
